# Global STEM education in the AI era

**DOI:** 10.1093/nsr/nwag564

**Published:** 2026-09-01

**Authors:** Mu-ming Poo

**Affiliations:** Scientific Director, CAS Center for Excellence in Brain Science & Intelligence Technology, China

## Abstract

Recent AI advances offer transformative opportunities for STEM education, but also could have negative impacts on the students’ brains and minds.

The establishment of the International Institute for STEM Education (IISTEM) in Shanghai in September 2025 marks a pivotal moment in global education in science, technology, engineering, and mathematics (STEM). As the first UNESCO’s Category 1 institute in China, IISTEM arrives at a critical juncture, when China is playing a leading role in global technological and economic development, including artificial intelligence (AI) applications in education. The IISTEM aims to become a center for global research collaborations on innovative educational approaches and a hub for resource sharing and capacity development in STEM education, especially in developing countries. It will also guide and validate the use of human-centered AI educational technologies.

Recent AI advances such as large language models (LLMs) offer transformative opportunities for STEM education. Personal AI chatbots could provide on-demand self-paced tutoring, especially in schools with staff shortage and student heterogeneity. Virtual labs can partially replace missing physical laboratories in resource-constrained schools. Appropriately designed software can assist students in analyzing, interpreting, and reporting experimental data. Generative AI tools could propose constraint-based engineering design alternatives, fostering iterative learning and innovative ideas. Instructors could use AI agents to help with designing curricula and preparing teaching materials.

However, there is also the serious issue that extensive AI use could have negative impacts on the students’ brains and minds. The information derived from AI may be inaccurate and biased, and students should learn how to validate and critically evaluate the information. Over-reliance on AI tools could undermine students’ academic integrity, critical thinking, reasoning, and analytical skills. Concerns about ill-adaptive neural plasticity need to be investigated: prolonged exposure to virtual–reality environments with incomplete sensory stimuli may impair the brain’s ability for multisensory processing in real-world scenarios, while persistent use of interactive AI tools, due to instant rewards these tools offer, could alter dopaminergic neural circuits in a manner resembling drug addiction. Thus, safeguards are required to balance the benefits and negative impacts of AI utility.

The accelerating pace of AI development also suggests the potential arrival of artificial general intelligence that is capable of learning, reasoning, and general problem-solving at a level equal to or surpassing that of humans, leading to drastic changes in the professional environments facing STEM graduates in coming decades. To enable the students to flourish in the AI era, AI proficiency training must become an integral part of STEM education, and emphasis should shift from specialty training towards multidisciplinary education and from disseminating existing knowledge towards cultivating students’ self-learning ability for acquiring new knowledge and skills. Notably, recent LLMs have acquired some cognitive/analytical abilities that approach those of humans, but largely lacking the affective/social intelligence, including empathy, ethics/morality, prosocial behaviors, and purpose setting. These capabilities represent the last bastion of humanity and should be nurtured in STEM education. Meanwhile, as humanoid robots are being developed, affective/social intelligence with empathy, ethics, and prosocial morality should become an essential part of their pre-training and the guardrails for their human alignment.

Some insights on how education may impart ethics and social values could be derived from Chinese education philosophy. Its three basic elements, as articulated by Han Yu (韩愈) 12 centuries ago, remain highly relevant today—disseminating ‘Dao’ (传道), teaching skills (授业), and dispelling doubts (解惑). The first element is particularly noteworthy, because ‘Dao’ comprises not only knowledge but also ethics and values, including justice, self-discipline, and the individual’s duty to society. This philosophy of ‘Dao’ has served as a pillar for Chinese civilization and a guiding principle underlying the Chinese view of the world. It was the ethics and social values of STEM graduates that empowered China’s remarkable scientific and technological (S&T) development in the past decades.

An ultimate goal of global STEM education is to nurture scientists and engineers who can address urgent issues facing all societies, such as climate change, environmental degradation, energy and food insecurity, infectious and chronic diseases, and the disparate distribution of S&T dividends among and within nations. Thus, an important task of IISTEM, besides promoting innovative approaches in teaching traditional STEM contents, is to develop curricula and teaching materials that integrate STEM education with humanities and social studies, such as ethics-related case studies in engineering design courses and studies based on AI-simulated scenarios of social dilemmas. Such integrative studies may help to nurture STEM students’ ethics/social concerns and commitment to solving critical issues of the world. The IISTEM, with its global mandate and location in a leading AI-powered nation, is uniquely positioned to lead this necessary educational transformation.

